# Time savings – realized and potential – and fair compensation for community health workers in Kenyan health facilities: a mixed-methods approach

**DOI:** 10.1186/1478-4491-13-6

**Published:** 2015-01-30

**Authors:** Laura D Sander, David Holtzman, Mark Pauly, Jennifer Cohn

**Affiliations:** Johns Hopkins Bloomberg School of Public Health, 1000 E. Eager Street, Baltimore, MD 21202 USA; Baylor International Pediatrics AIDS Initiative, Maseru, Lesotho; University of Pennsylvania Wharton School, Philadelphia, PA USA; Division of Infectious Diseases, University of Pennsylvania School of Medicine, Philadelphia, PA USA

**Keywords:** task shifting, community health workers, Kenya, compensation

## Abstract

**Background:**

Sub-Saharan Africa faces a severe health worker shortage, which community health workers (CHWs) may fill. This study describes tasks shifted from clinicians to CHWs in Kenya, places monetary valuations on CHWs’ efforts, and models effects of further task shifting on time demands of clinicians and CHWs.

**Methods:**

Mixed methods were used for this study. Interviews were conducted with 28 CHWs and 19 clinicians in 17 health facilities throughout Kenya focusing on task shifting involving CHWs, time savings for clinicians as a result of task shifting, barriers and enabling factors to CHWs’ work, and appropriate CHW compensation. Twenty CHWs completed task diaries over a 14-day period to examine current CHW tasks and the amount of time spent performing them. A modeling exercise was conducted examining a current task-shifting example and another scenario in which additional task shifting to CHWs has occurred.

**Results:**

CHWs worked an average of 5.3 hours per day and spent 36% of their time performing tasks shifted from clinicians. We estimated a monthly valuation of US$ 117 per CHW. The modeling exercise demonstrated that further task shifting would reduce the number of clinicians needed while maintaining clinic productivity by significantly increasing the number of CHWs.

**Conclusions:**

CHWs are an important component of healthcare delivery in Kenya. Our monetary estimates of current CHW contributions provide starting points for further discussion, research and planning regarding CHW compensation and programs. Additional task shifting to CHWs may further offload overworked clinicians while maintaining overall productivity.

## Background

There is an estimated deficit of 2.4 million doctors, nurses and midwives globally. The majority of this deficit lies in sub-Saharan Africa, which accounts for 24% of the global burden of disease yet has only 3% of the world’s healthcare workers (HCWs) [1]. Determining how to expand the healthcare workforce and enhance its quality is necessary for affected countries to achieve their health-related millennium development goals (MDGs) [1–4]. Task shifting - the reassignment of clinical roles by transferring suitable tasks from higher- to lower-skilled HCWs - is one of the strategies proposed to mitigate the effects of the HCW shortage.

Community health workers (CHWs) are a unique cadre of HCWs and are well suited to help address the HCW gap. Many studies have demonstrated that CHWs can be as effective as more highly skilled HCWs in delivering a variety of services [5–14] and help achieve MDGs [15]. But until recently, there have been few large-scale, sustainable CHW programs implemented in developing countries. Known barriers to successful implementation of CHW programs include inadequate compensation of CHWs, frequent unreimbursed out-of-pocket expenditures and variability in quality and duration of training and supervision [16–20].

Kenya’s 2006 Community Strategy (CS) outlines primary health care delivery at the community unit (CU). CUs comprise volunteer CHWs who are linked to the primary health facility through trained Community Health Extension Workers (CHEWs) employed in primary care facilities; each CHEW is meant to supervise 25 CHWs [21]. CHWs are men and women recruited from the community, ‘having demonstrated attitudes valued by the community’ , and, preferably, are literate [21]. Main tasks performed by CHWs are categorized as: 1) disease prevention and control, 2) family health services, and 3) hygiene and environmental sanitation [22]. Further, the CS stipulates that volunteer CHWs should be paid a stipend ‘based on work actually done’ [21]. In a 2011 telegram, Kenya’s Ministry of Public Health and Sanitation stated ‘where funds are available community health workers shall be entitled to a payment of Ksh.2000 per month [approximately USD$ 25] as performance based incentive’ [23]. This statement lacks guidance on where funds should come from and on standardized criteria for performance standards. It remains unclear if and how many CHWs are compensated for their work.

Clinical officers and nurses are also integral to health care at the CU. Clinical officers undergo at least 3 years of university-level training and a year of internship, providing patient care and management at the primary health care level [24, 25]. Nurses undergo 1 to 4 years of training and, accordingly, achieve certificates, diplomas or degrees [26]. Kenya is facing a severe HCW shortage, and attrition among clinical officers is particularly high at the dispensary level [27, 28]. In 2010, the average monthly wage for clinical officers was USD$ 372, for nurses it was USD$ 248, and for public health officers it was USD$ 124 (J. Mwitari, personal communication, March 2010).

In practice, diverse and distinct community health models have emerged since 2010 when Kenya decentralized health care administration [29]. Faith-based and civil society organizations have played an important role in expanding the government’s efforts to establish CUs [30]. As Kenya works toward its One Million Community Health Workers Campaign to standardize CUs, it is important to detail tasks that volunteer CHWs perform. In addition, no studies have looked at the actual time savings realized by task shifting from higher HCW cadres to CHWs [18]. This information is needed to inform implementation of task shifting and help developing country governments, donors and non-governmental organizations (NGOs) better understand the value of CHWs. This mixed-methods study in Kenya was conducted to fill this knowledge gap.

## Methods

### Study setting and population

This was a collaborative, mixed-methods study between the University of Pennsylvania and GROOTS Kenya, a community-based organization that supports CHWs throughout Kenya. Semistructured interviews (qualitative) and task diaries (quantitative) were used to triangulate and develop valuation of CHW tasks; both methods were then used to develop and initiate clinic modeling, the second quantitative component [31].

Seventeen health facilities within the GROOTS network were chosen using quota sampling based on the population densities of the community. Based on demographic data, health facilities were categorized to be in densely, mid- or sparsely populated areas [32, 33]. More facilities in sparsely populated areas were selected since the majority of Kenyans reside in rural settings. Facilities included dispensaries, health centers and district hospitals. Site visits occurred between October 2010 and March 2011.

The leader of each health facility was asked to identify 1 to 3 clinical officers and/or nurses and 1 to 3 CHWs for possible enrolment in the study. Potential subjects were approached and informed consent obtained. Study participants were required to be predominately facility-based and are distinguished from CHEWs, as the latter supervise CHWs and do not directly conduct health-related tasks. CHWs are therefore defined as a primary health worker caring for community members. In addition, they are volunteers: monies received for services were largely applied toward procurement of supplies or transportation to outreach community members and also were typically not consistent. Furthermore, monies came from associated NGOs, and not the Ministry of Health. The University of Pennsylvania Institutional Review Board approved the study.

### Semistructured interviews

Semistructured interviews were conducted with all study participants. Clinician interviews focused on 1) assessing tasks shifted from clinicians to CHWs, 2) estimating time freed by task shifting and 3) identifying additional tasks that could be shifted to CHWs. CHW interviews focused on 1) current tasks performed, 2) self-perception of their ability to perform these tasks, 3) views on additional tasks that could be shifted to CHWs and 4) additional support needed to improve performance and permit further task shifting. All participants were asked to estimate an appropriate monthly salary for local CHWs based on their current work. Interviews were recorded, transcribed, and analyzed using Microsoft Excel.

### Task diaries

Task diaries were distributed to CHWs to complete over a consecutive 14-day period. The task diary contained a list of common CHW tasks developed by GROOTS Kenya administrators and representative CHWs, and provided space for additional tasks to be entered. Each day CHWs were asked to record every task performed, the number of patients reached with each task, and the amount of time spent per task. Diary data were analyzed using Microsoft Excel.

### Analysis of semistructured interviews and task diaries

Two research staff (LS and DH) conducted and analyzed the semistructured interviews. Tasks recorded in the CHW diaries were categorized as a directly-shifted or added-value task. Directly-shifted tasks were ‘core services’ traditionally performed by clinicians (for example, patient registration, measuring vital signs and administering immunizations). All other tasks were considered to be added-value services, which are generally not provided unless a CHW is available (for example, providing individual and group education and counseling, mobilizing communities). Categorization of tasks was based on consensus of clinicians and CHWs interviewed, and are displayed in Table 1. The total and proportional amount of time spent performing each task was calculated for every CHW and aggregated by category. Data were analyzed by population density and facility type.

**Table 1:** **List of clinic services**

Core services (services generally provided)	Added value services (services generally not provided unless a CHW is available)
Patient registration	Group health education talks
Billing	Community mobilization
Time with clinician	Social work support
Filing	Nutrition support
Measuring vital signs*	Defaulter tracing
Basic and intensive adherence monitoring and counseling*	Linking health facility and community
Lab services*	
Immunizations*	
Medication and supply distribution*	
HIV voluntary counseling and testing*	

*Denotes ‘directly-shifted’ services designated by clinical officers, nurses and CHWs as tasks traditionally performed by clinicians that could be shifted to CHWs. CHW, community health worker.

The monetary value of time CHWs spent performing directly-shifted tasks was estimated using wage data for public health officers, clinical officers and nurses obtained by personal interview with the Ministry of Public Health and Sanitation in March 2010 (J. Mwitari, personal communication, March 2010). Since nurses provide most clinical services at dispensaries while clinical officers do so in hospital-linked clinics, nurse salary was used for dispensaries and clinical officer salary for hospital clinics. An average of clinical officer and nurse salaries was used for health centers. Clinicians and CHWs perceived CHWs to be less efficient than clinicians, likely a result of inadequate CHW training. Accordingly, the time a CHW spent performing directly-shifted tasks was reduced by a correction factor of two-thirds, based upon discussions with clinicians and CHWs during the interviews. This adjusted time was multiplied by the salary figure corresponding to the facility type where the CHW worked to arrive at an estimate of a lower bound monetary value of time each CHW spent on directly-shifted tasks.

Since CHWs are not typically compensated, we had no direct way of valuing the added-value services they provide. Hence, assumptions about the intrinsic value of this work were made. We felt that the work of a CHW was commensurate with a public health officer, and learned the average monthly wage of that cadre of health workers is USD$ 124 per month. This figure is commensurate with the average wage suggested by clinicians in semistructured interviews (see Results), and we felt would be a good starting point by which to value the CHWs’ added-value services. This wage was multiplied by the time each CHW spent performing added-value tasks to arrive at a valuation of these services. All analyses were conducted using Microsoft Excel.

### Modeling

**Figure 1:**
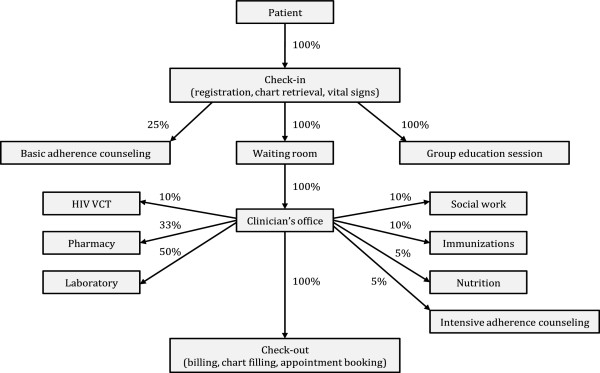
**Model clinic flow.** A model clinic was created to examine the effects of shifting various tasks from clinicians to community health workers. The figure depicts the progression of patients through a clinic visit and highlights some of the various services they may require. Percentages indicate the estimated proportion of patients requiring that service, based on interviews with clinicians and the authors’ clinical experience working in Kenyan health facilities. Table 2 displays all assumptions that were used to create the model clinic.

A model clinic was created to examine the effects of shifting various tasks from clinicians to CHWs on the time demands of clinicians and CHWs. Figure 1 depicts the progression of patients through a clinic visit and highlights some services patients may require.

The model was created using Microsoft Excel with the following primary inputs: 1) number of patients seen each day at the clinic, 2) number of patients seen each day by one clinician, 3) the proportion of patients requiring a service, and 4) the amount of time to complete each service. Patient volumes were derived from interviews with clinicians. Table 2 outlines all of the inputs for model assumptions, which were based on the authors’ clinical experience working in Kenyan health facilities.

**Table 2:** **Assumptions used to develop the model clinic**

Task	Percentage/Number of patients requiring service	Average time to complete service per patient (minutes)	Cadre currently performing task	Cadre ideally performing task
Check-in (registration, chart retrieval, vitals)	100%	3	Clerk	Clerk
Vital signs measurement	100%	3	Clinician	CHW
Clinician visit	100%	7	Clinician	Clinician
Lab services	50%	5	Clinician	CHW
Medication and supply distribution	33%	5	Clinician	CHW
Immunizations	10%	5	Clinician	CHW
Basic adherence counseling	25%	5	Clinician	CHW
Intensive adherence counseling	5%	10	Clinician	CHW
Nutrition support	5%	15	Various	CHW
Social work support	10%	15	Social work	Social work
HIV voluntary counseling and testing	10%	10	Various	CHW
Check-out (billing, chart filing, appointment booking)	100%	5	Clerk	Clerk
Group education session	100%	30	CHW	CHW
Defaulter tracking	2	30	CHW	CHW
Community mobilization	--	60	CHW	CHW
Linking facility and community	--	60	CHW	CHW
Patients seen per clinician per day	25	--	--	--
Patients attending clinic per day	35	--	--	--

A determination was then made for each service based on the information from clinician and CHW interviews regarding which cadre currently performs the service and which cadre would ideally perform it so as to best utilize health worker skills (Table 2). The main tasks targeted for task shifting are denoted by ‘*’ in Table 1.

The time demands for CHWs and clinicians were then calculated for these ‘current’ and ‘idealized’ task allocation scenarios. A sensitivity analysis was performed in which the amount of time required for each cadre to complete a service was varied by a factor of two to determine the effects of these assumptions in the model.

## Results

Eight locations incorporating 17 health facilities (7 hospitals, 6 health centers and 4 dispensaries) were visited in Nairobi, Nyanza, Central, Eastern and Western Provinces. Ten sites were in sparsely populated areas, three in mid-populated regions and four in densely populated areas.

Forty-seven interviews were conducted with 28 CHWs and 19 clinicians. No potential participant declined to be interviewed. All 28 CHWs interviewed agreed to complete the task diaries; 20 diaries were returned; and 3 were not included in the analysis due to incorrect recording of time spent performing tasks. Overall, the CHWs spent 60% of their time in facilities and 40% of their time in the community. Tasks performed in the community include defaulter tracing and linking community members to the health facility. Counseling and health education occurred both in the community and facility; all other tasks occurred in the facility.

### Semistructured interviews

CHWs reported performing a variety of tasks (Table 3). For each of these tasks, clinicians also were asked if these tasks have been shifted from their duties. CHWs and clinicians report varying levels of task shifting. CHWs provided an important link between health facilities and communities by performing defaulter tracing, home visits and outreach education; interviewees highlighted this link as previously missing or inadequate. The majority of CHWs performed tasks that have been shifted from other cadres, including health education, counseling, taking vital signs, and dressing simple wounds. CHWs and clinicians both cited the need for additional training, reliable provision of supplies, consistent and adequate compensation, and transportation support in order to improve the work of CHWs to enable them to take on more tasks and to retain them.

**Table 3:** **Tasks performed by community health workers (CHWs) and clinician-reported status of task shifting**

Tasks	CHWs currently performing task (%), n = 28	Clinicians reporting task as shifted (%), n = 19
Register patients	17 (61%)	12 (63%)
Take vital signs	18 (64%)	8 (42%)
Dispense medications	9 (32%)	5 (26%)
Provide individual education/counseling	22 (79%)	8 (42%)
Provide group education	21 (75%)	16 (84%)
Community mobilization	18 (64%)	7 (37%)
Linking health facility and community	10 (36%)	7 (37%)
Defaulter tracing	19 (68%)	16 (84%)

Clinicians estimated that CHWs save the clinicians an average of 2.5 hours of work per day (SD 1.1 hours). The average monthly salary estimated by interviewees to be fair compensation for the CHWs’ current work was US$ 160 (SD US$ 110); CHWs provided a higher estimate than clinicians (US$ 182 for CHWs, US$ 128 for clinicians).

### Task diaries

Table 4 presents data from the CHW task diaries on the number of hours CHWs spent performing core services and added-value services. CHWs worked an average of 5.3 hours/day (SD 2.5 hours) with 36% of their time spent on directly-shifted tasks and 64% on added-value work. On average, CHWs worked 12 of the 14 days surveyed (range 9 to 14 days). CHWs in rural areas and those based at dispensaries worked more hours per day, mostly due to a greater amount of time spent performing added-value tasks.

**Table 4:** **Current community health worker (CHW) time contributions by facility type and population density**

		Average number of days worked in 14 day period by CHWs	Core Services		Added value services		All services	
			Avg hrs/day (% total)	Total hours	Avg hrs/day (% total)	Total hours	Avg hrs/day	Total hours
Facility type								
Hospital	11	12.1	2.2 (42%)	297	3.1 (58%)	418	5.4	715
Health center	4	13.0	1.4 (35%)	74	2.6 (65%)	134	4.0	208
Dispensary	2	14.0	1.3 (16%)	35	6.5 (84%)	181	7.7	216
Population density								
Densely populated	5	13.6	2.0 (49%)	135	2.1 (51%)	143	4.1	277
Mid-populated	4	11.8	2.0 (31%)	93	4.4 (69%)	206	6.4	299
Sparely populated	8	12.3	1.8 (32%)	179	3.9 (68%)	384	5.7	563
Total	17	12.5	1.9 (36%)	406	3.4 (64%)	733	5.3	1139

Table 5 displays the valuations of the CHW work recorded in the task diaries. The 17 CHWs surveyed worked 1,139 hours valued at US$ 999 (US$ 0.88 per hour). Directly-shifted and added-value tasks accounted for nearly equal parts of this value: US$ 459 (46%) and US$ 540 (54%), respectively. The average value of each CHW’s work per 14-day period was US$ 59 (range US$ 45 to 79). Averaged over a year, this represents a monthly salary of US$ 117; if the average hourly CHW wage of US$ 0.88 and a 40-hour workweek are used, the projected monthly salary is US$ 147.

**Table 5:** **Monetary estimates of community health worker (CHW) contributions by facility type and population density for a 14-day study period and per month (all values in US$)**

	CHWs	Directly shifted value		Added value		Total value		
		Total*	Per CHW*	Total	Per CHW	Total	Per CHW	Projected monthly value
Facility type								
Hospital	11	$ 357	$ 32	$ 311	$ 28	$ 668	$ 61	$ 121
Health center	4	$ 80	$ 20	$ 100	$ 25	$ 180	$ 45	$ 90
Dispensary	2	$ 26	$ 13	$ 135	$ 67	$ 160	$ 80	$ 160
Population density								
Densely populated	5	$ 149	$ 30	$ 106	$ 21	$ 255	$ 51	$ 102
Mid-populated	4	$ 105	$ 26	$ 153	$ 38	$ 258	$ 64	$ 1
Sparsely populated	8	$ 210	$ 26	$ 286	$ 36	$ 496	$ 62	$ 124
Total	17	$ 463	$ 27	$ 545	$ 32	$ 1008	$ 59	$ 119

*Discounted for assumed time inefficiency of a CHW completing a task compared to a clinician.

### Modeling

Clinician interviews revealed an average load of 35 patients per clinician per day (range 10 to 40). Using the model described (Figure 1 and Table 2) with the specified inputs, the average time for an individual patient clinic visit is 40 minutes, not including waiting time; thirty-three minutes of this time is accounted for by direct patient care and the remainder by general clinic services that indirectly benefit patients. The minimum amount of time for a clinic visit was 26 minutes and the maximum was 1 hour and 56 minutes.

Table 6 presents the work hours required by each HCW cadre to provide services to 35 patients per day in current and in idealized scenarios. This model projects that additional task shifting measures would free an estimated 6.1 hours per day of clinician time while adding 9.5 hours per day of CHW time, given that CHWs are less efficient at performing tasks than clinicians. If clinicians and CHWs were less efficient at performing tasks than assumed, it would have a greater effect on the potential time savings for clinicians than if they were more efficient.

**Table 6:** **Clinic model - effect of task shifting on health cadre work hours (based on 35 patient visits per day)**

	Current scenario ^a^ (hours per day)			Ideal scenario ^b^ (hours per day)			Difference ^c^ (hours per day)		
Health cadre	More efficient	Baseline	Less efficient	More efficient	Baseline	Less efficient	More efficient	Baseline	Less efficient
Clinician	4.7	10.2	19.8	1.8	4.1	8.2	−2.9	−6.1	−11.6
CHW	6.3	9.3	14.5	11.0	18.8	33.2	+4.7	+9.5	+18.7
Total	9.3	19.5	38.5	11.7	22.9	47.3	+1.8	+3.4	+7.1

This table presents work hours required by each healthcare worker to provide services to 35 patients per day in current and idealized scenarios. The amount of time required for each cadre to complete a service was varied by a factor of two, representing more and less efficiency, to determine the effects of these assumptions in the model. In the baseline current scenario,^a^ clinicians work 10.2 hours per day while CHWs work 9.3 hours per day. In a baseline ideal scenario,^b^ task shifting would result in clinicians working 4.1 hours per day while CHWs would work 18.8 hours per day. With appropriate tasks shifted to community health workers,^c^ 6.1 hours of clinician time would be freed, while 9.5 hours of community health worker time would be added. If healthcare workers were less efficient at performing tasks, this would have a greater effect on potential time savings than if they were more efficient. CHW, community health worker.

Using this model, a health facility servicing 35 patients per day would require 0.5 clinician full-time equivalents (FTEs) and 2.4 CHW FTEs based on an 8-hour workday if the selected additional tasks were shifted. This is opposed to 1.3 clinician FTEs and 1.2 CHW FTEs required without further task shifting. The current scenario would cost US$ 470 to 631 per month for wages depending on the type of facility and assuming a monthly CHW salary of US$ 124, which is a reasonable figure suggested from this study. The idealized scenario would cost an estimated US$ 421 to 545 per month in wages, a 10.4 to 13.6% reduction in monthly wages for the same expected overall clinic productivity and would require fewer clinicians overall.

## Discussion

Multiple studies and a recent meta-analysis support that lay HCWs can be as effective as higher skilled HCWs at providing a wide range of services [8–11, 34]. In Kenya, CHWs providing care for antiretroviral drug delivery had similar outcomes of HIV control compared to standard clinic visits [12]. Our study’s findings are consistent with these studies in that semistructured interviews and CHW task diaries demonstrated that facility-based CHWs are delivering a significant amount and variety of services, including tasks that have been directly-shifted from clinicians as well as those that are added-value to Kenya’s health system. Tasks performed and the amount of task shifting varies among CHWs and as perceived by clinicians. It is important to note our study did not objectively measure quality of care provided by CHWs, however. In addition, we were unable to consider additional important aspects of the CHW workforce, including attrition, training and supervision.

A study in Rwanda on task shifting from physicians to nurses showed a 78% reduction in HIV-related physician workload as a result of implementing nurse-initiated antiretroviral therapy, saving up to 56 hours of physician time per month [35]. Our interviews revealed that task shifting to CHWs frees up 2.5 hours/day of clinicians’ time on average. This is the first published data that we know of to quantify the time savings realized through CHW task shifting. In addition, our modeling exercise suggests transitioning other selected tasks from clinicians to CHWs could yield further significant time savings for clinicians while maintaining clinic productivity. These findings are consistent with studies showing cost-effectiveness of CHW-based care [5–7, 13, 36, 37].

Prior research has identified the lack of consistent and appropriate remuneration of CHWs as a significant barrier to the success of CHW programs. Paying CHWs appropriately for their work and providing clear delineation of CHW tasks has been shown to enhance CHW retention and improve sustainability of CHW programs. [2, 7, 18, 38–40] While a recent study enumerated health system-level costs associated with deploying CHWs using an estimate of current CHW wages [41], we attempted to quantify the monetary value of facility-based CHWs’ work to provide concrete starting data points for the discussion of fair CHW compensation.

CHWs provided higher estimates of a fair monthly salary than clinicians (US$ 178 and US$ 128, respectively). Using the data from the CHW task diaries, we derived an average monthly valuation of current CHW services of US$ 117, consistent with the clinicians’ estimated CHW wage. While this calculation includes added value of CHWs it is likely an underestimate because we were not able to quantify important linkages that CHWs provide to health facilities.

Our modeling exercise suggests that further HCW skill optimization through additional task shifting and the expansion of CHW roles could justify employing 2.5 CHWs at an average-sized health facility at current workloads. Our clinic model is limited by the assumptions made regarding the average completion time for tasks; the proportion of patients in need of each service; the cadre that currently performs a task and which cadre would ideally do it; and the extra time required to complete a task after it has been shifted to a CHW. These assumptions affect the time required of each HCW to provide their services to an individual patient and the total daily cadre times. The sensitivity analysis showed significant variability in the projected clinician time savings and additional CHW time. This highlights the need for future research on these variables in resource-limited clinics in order to better inform models.

Further, in Kenya, CHEWs are facility-based HCWs, but are meant to take on mostly a supervisory role of CHWs. As additional models of CHEWs and CHWs are developed, it may be necessary to create complementary cadres of CHWs: those that are predominately facility-based and take on mostly task-shifted roles in the facility, and those that are community-based and engage mostly in added-value services that CHWs provide in the community.

There are several limitations to our estimates of the value of CHWs’ work. Study subjects may have been more strongly supportive of CHWs given that all who were approached agreed to participate; this selection bias may have influenced results. Misclassification of tasks may also have occurred as they were categorized as directly-shifted or added-value based on semistructured interviews. The valuation of directly-shifted CHW tasks was affected by the uniform time adjustment made for the assumption that clinicians are more efficient than CHWs; there is, however, likely significant variability in the relative efficiency of CHWs and clinicians. The research team chose the value of this discount factor based on input from clinicians and CHWs and their own experiences, not objective data due to an absence of relevant published data. The valuation of added-value tasks was based on a wage figure derived from our interviews with CHWs and clinicians. Because of this linkage, our valuation is less valid than if we had used an estimated CHW salary obtained by other means. CHWs had an incentive to provide a higher estimated CHW wage if they felt they stood to benefit from any future CHW compensation program, although CHWs may also have a better sense of what might be a fair living wage. Lastly, our estimate of clinician time liberated is limited since we did not simultaneously record clinicians’ daily tasks. Nonetheless, we believe our estimates provide useful reference points for policy debates and future research.

In summary, CHWs are an important component of health service delivery in Kenya. They reduce the workload of clinicians, giving them more time to spend with patients, see additional patients and do higher-level job functions. They also provide services that would often not be provided otherwise and provide vital links between communities and health facilities. Our study attempts to put a value on these services to aid those involved with planning, financing, and implementing CHW programs in resource-limited settings. While compensation is just one component of the CHW programs that exist within complex parent health systems, it is important to appropriately address it upfront as these programs are designed, implemented, and scaled up.

## Conclusions

CHWs are an important component of healthcare delivery in Kenya. The estimates herein provide starting points for fair compensation of CHWs, so they may be further integrated into health systems faced with a health worker shortage. Optimal utilization of CHWs will reduce the workload of strained health workers and improve access to health care.

## Abbreviations

CHEWs: community health extension workers
CHWs: community health workers
CS: community strategy
CU: community unit
FTE: full-time equivalents
HCWs: healthcare workers
MDGs: millennium development goals
NGOs: nongovernmental organizations.

## References

[1] World Health Organization (2006). Working together for health. In World health report 2006.

[2] World Health Organization (2008). Task shifting: rational redistribution of tasks among health workforce teams: global recommendations and guidelines.

[3] Samb B, Celletti F, Holloway J, Van Damme W, De Cock KM, Dybul M (2007). Rapid expansion of the health workforce in response to the HIV epidemic. N Engl J Med.

[4] Chen L, Evans T, Anand S, Boufford JI, Brown H, Chowdhury M (2004). Human resources for health: overcoming the crisis. Lancet.

[5] Islam A, Wakai S, Ishikawa N, Chowdhury A, Vaughan J (2002). Cost-effectiveness of community health workers in tuberculosis control in Bangladesh. Bull World Health Organ.

[6] Nganda B, Wangombe J, Floyd K, Kangangi J (2003). Cost and cost-effectiveness of increased community and primary care facility involvement in tuberculosis care in Machakos District Kenya. Int J Tuberc Lung Dis.

[7] Walker DG, Jan S (2005). How do we determine whether community health workers are cost-effective? Some Core Methodological Issues. J Community Health.

[8] Weidle PJ, Wamai N, Solberg P, Liechty C, Sendagala S, Were W (2006). Adherence to antiretroviral therapy in a home-based AIDS care programme in rural Uganda. Lancet.

[9] Morris MB, Chapula BT, Chi BH, Mwango A, Chi HF, Mwanza J (2009). Use of task-shifting to rapidly scale-up HIV treatment services: experiences from Lusaka, Zambia. BMC Health Serv Res.

[10] Callaghan M, Ford N, Schneider H (2010). A systematic review of task-shifting for HIV treatment and care in Africa. Hum Resour Health.

[11] Bemelmans M, van den Akker T, Ford N, Philips M, Zachariah R, Harries A (2010). Providing universal access to antiretroviral therapy in Thyolo, Malawi through task shifting and decentralization of HIV/AIDS care. Trop Med Int Heal.

[12] Selke HM, Kimaiyo S, Sidle JE, Vedanthan R, Tierney WM, Shen C (2010). Task-shifting of antiretroviral delivery from health care workers to persons living with HIV/AIDS: clinical outcomes of a community-based program in Kenya. J Acquir Immune Defic Syndr.

[13] Datiko D, Lindtjorn B (2010). Cost and cost-effectiveness of smear-positive tuberculosis treatment by health extension workers in southern Ethiopia: a community randomized trial. PLoS One.

[14] Mwai GW, Mburu G, Torpey K, Frost P, Ford N, Seeley J (2013). Role and outcomes of community health workers in HIV care in sub-Saharan Africa: a systematic review. J Int AIDS Soc.

[15] Perry H, Zulliger R (2012). How effective are community health workers?.

[16] Republic of Kenya Ministry of Public Health and Sanitation (2009). Report of the review of the implementation of the community health strategy.

[17] Partnership The United National Development Program Japan and GROOTS Kenya (2009). Compensation for contribution report: a baseline study.

[18] Lehmann U, Sanders D (2007). Community health workers: What do we know about them?.

[19] Hermann K, Van Damme W, Pariyo GW, Schouten E, Assefa Y, Cirera A (2009). Community health workers for ART in sub-Saharan Africa: learning from experience–capitalizing on new opportunities. Hum Resour Health.

[20] Jaskiewicz W, Tulenko K (2012). Increasing community health worker productivity and effectiveness: a review of the influence of the work environment. Hum Resour Health.

[21] Republic of Kenya Ministry of Health (2006). Taking the Kenya Essential Package for Health to the Community: A Strategy for the Delivery of Level One Services.

[22] Mireku M, Kiruki M, McCollum R, Taegtmeyer M, de Koning K, Otiso L (2014). Context Analysis: close-to-community health services providers in Kenya.

[23] Republic of Kenya Ministry of Public Health and Sanitation (2011). 8 March 2011). Policy shift on community strategy (Ref. No: MPHS/ADM/2/30 Vol.IV.

[24] Government of Kenya (2010). Revised scheme of service for clinical officers.

[25] Mbindyo P, Blaauw D, English M (2013). The role of Clinical Officers in the Kenyan health system: a question of perspective. Hum Resour Health.

[26] Nursing Council of Kenya (2014). Education Information: Approved Nursing Programmes.

[27] Chankova S, Muchiri S, Kombe G (2009). Health workforce attrition in the public sector in Kenya: a look at the reasons. Hum Resour Health.

[28] World Health Organization (2010). Global Atlas of the Health Workforce.

[29] One Million Community Health Workers Campaign (2014). Update from the field: Kenyan community health services works to establish national standards.

[30] Government of Kenya and UNICEF (2010). Evaluation report of the community health strategy implementation in Kenya.

[31] Greene JC, Caracelli VJ, Graham WF (1989). Toward a conceptual framework for mixed-method evaluation designs. Educ Eval Policy Anal.

[32] Columbia University Center for International Earth Science Information Network (2013). Gridded population of the world.

[33] Kenya National Bureau of Statistics (2015). Country stats.

[34] Lassi ZS, Gometto G, Huicho L, Bhutta ZA (2013). Quality of care provided by mid-level health workers: systematic review and meta-analysis. Bull World Health Organ.

[35] Chung J, O’Brien M, Price J, Shumbusho F, International AIDS Society: *Quantification of physician-time saved in a task shifting pilot program in Rwanda. AIDS 2008 – XVII Int*. Geneva, Switzerland: AIDS Conference; . Accessed 16 May 2011 http://www.aids2008.org/Pag/Abstracts.aspx?SID=255&AID=6043

[36] Lewin S, Munabi-Babigumira S, Glenton C, Daniels K, Bosch-Capblanch X, van Wyk BE (2010). Lay health workers in primary and community health care for maternal and child health and the management of infectious diseases. Cochrane Database Syst Rev.

[37] Hongoro C, McPake B (2004). How to bridge the gap in human resources for health. Lancet.

[38] Schneider H, Hlophe H, van Rensburg D (2008). Community health workers and the response to HIV/AIDS in South Africa: tensions and prospects. Health Policy Plan.

[39] Philips M, Zachariah R, Venis S (2008). Task shifting for antiretroviral treatment delivery in sub-Sahran Africa: not a panacea. Lancet.

[40] Nkonki L, Cliff J, Sanders D (2011). Lay health worker attrition: important but often ignored. Bull World Health Organ.

[41] McCord GC, Liu A, Singh P (2013). Deployment of community health workers across rural sub-Saharan Africa: financial considerations and operational assumptions. Bull World Health Organ.

